# Spectral Analysis Methods Based on Background Subtraction and Curvature Calculation Used in the Detection or Quantification of Hemolysis and Icterus in Blood-derived Clinical Samples

**DOI:** 10.7759/cureus.1965

**Published:** 2017-12-19

**Authors:** Toan Huynh, Michael J Lai, Yang L Liu, Linda Ly, Xinwei Gong, Kathryn R Rommel, Daniel L Young

**Affiliations:** 1 Assay Development, Theranos, Inc.; 2 Computational Biosciences, Theranos, Inc.

**Keywords:** point-of-care, sample integrity, chemistry, spectroscopy, interfering substances

## Abstract

Objective

We aimed to find new methods to detect and quantify hemolysis and icterus which may cause assay biases. These methods need to determine each of these interferents in the presence of various other interferents. They also need to have less stringent requirements in development and implementation than those conventional analyzers currently must satisfy.

Design and methods

We developed two spectral analysis methods that obtain absorption signals of interest by background subtraction or by calculating the spectral curvatures near the peaks of interest. We optimized and tested the performance of these methods using a plasma sample set with permutations of the levels of hemolysis, icterus, and lipemia (using 510 samples in total).

Results

The processed signals correlated well with concentrations of hemoglobin and bilirubin, indicators of hemolysis and icterus, respectively. Through iterations of randomly splitting the samples for calibration and testing, the two new methods performed as well as those used on conventional analyzers. We demonstrated that the two methods can lessen the application requirements of 1) prior knowledge of the absorption spectra of individual interferents, 2) calibration over a wide concentration range for each interferent, and 3) the need for full-range spectrophotometers spanning most of the ultraviolet/visible spectrum. We also proposed a hardware setup to detect and quantify hemolysis or icterus with a camera and two optical filters.

Conclusions

This work indicates that new methods of spectral analysis can reduce practical constraints in the development of interference screening systems. These methods could also benefit other assays that rely on reading spectral signals.

## Introduction

It is necessary to quantify and detect hemolysis, icterus, and lipemia (common interferents) in plasma and serum clinical samples [1], as they can introduce assay biases, especially in clinical chemistry [2]. The detection and quantification of each of these interferents alone are straightforward but become complicated when multiple interferents are concurrently present. Traditional methods utilize absorbance values at multiple wavelengths to account for potentially interfering signals from other interferents. They require calibrations that span possible concentration ranges of these interferents and instruments that can measure absorbance values across the ultraviolet/visible wavelength range [1]. This paper describes two new methods to quantify and detect hemolysis and icterus (two of the three interferents) that have fewer requirements in development and implementation.

These two new methods involve calculating either the background-subtracted signals or curvatures from spectral data. The advantages of these new methods are three-fold: 1) the elimination of the need to know beforehand how other interferents affect the detection and quantification of the interferent being investigated, 2) fewer samples required for calibration, and 3) fewer constraints on hardware design (thanks to the narrower ranges of required wavelengths).

**Figure 1:**
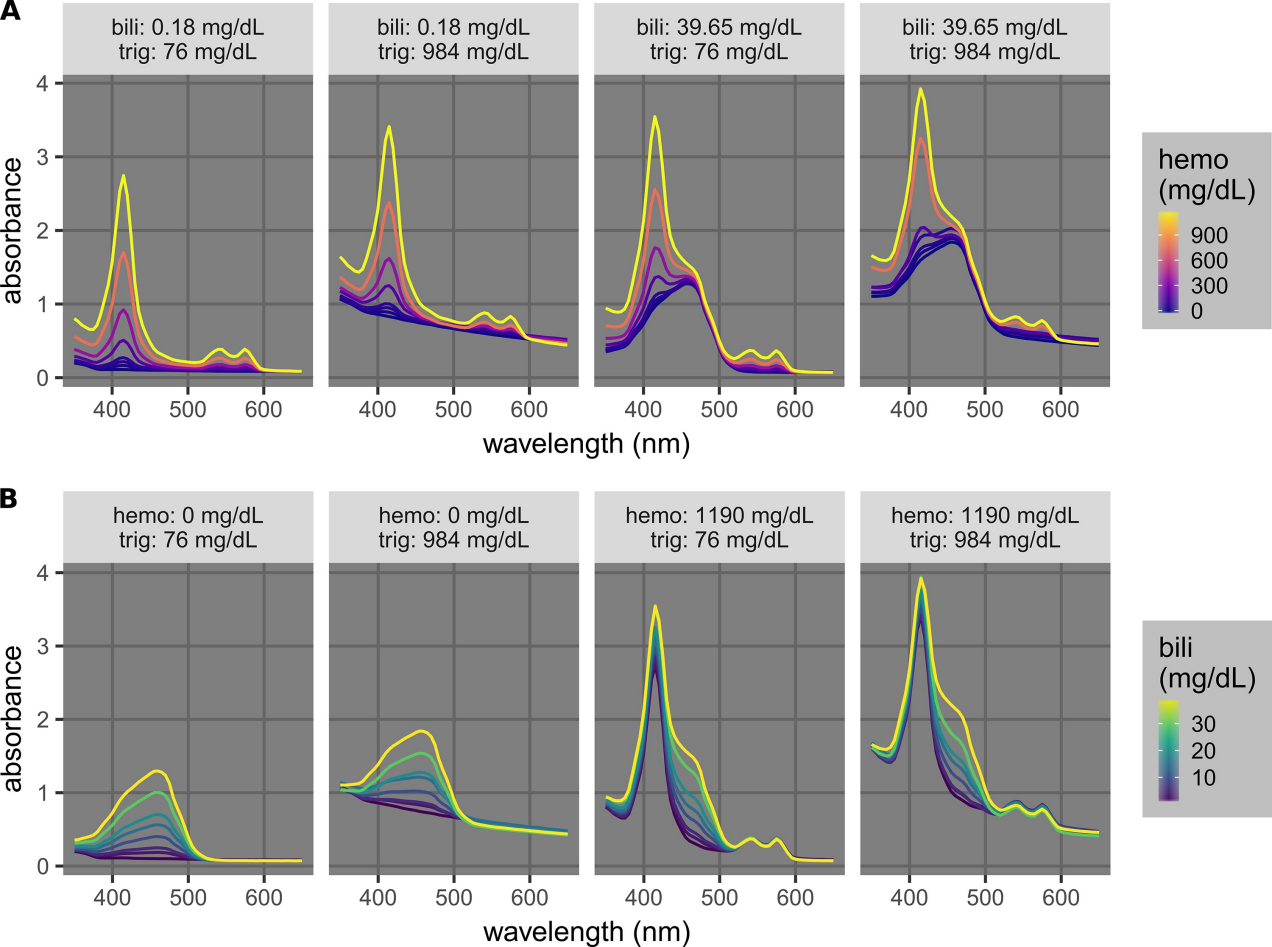
Complications of the quantification and detection of hemolysis in the presence of icterus and lipemia (A) and of icterus in the presence of hemolysis and lipemia (B). The levels of hemolysis, icterus, and lipemia are quantified by the concentrations of hemoglobin (hemo), bilirubin (bili), and triglycerides (trig), respectively. A) Spectra of samples with different hemo (0, 30, 50, 70, 180, 370, 760 and 1190 mg/dL) at low/high permutations of bili (0.18/39.65 mg/dL) and trig (76/984 mg/dL). B) Spectra of samples with different bili (0.18, 2.76, 4.77, 9.62, 14.63, 19.20, and 29.63 mg/dL) at low/high permutations of hemo (0/1190 mg/dL) and trig (76/984 mg/dL).

**Figure 2:**
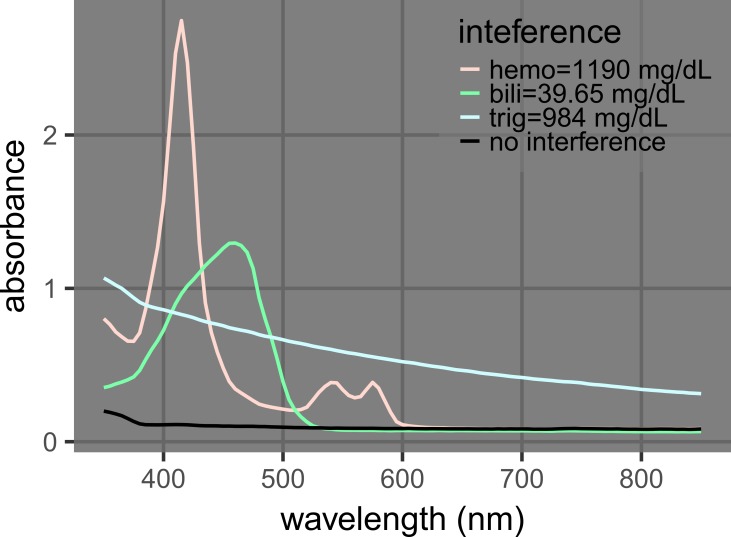
Spectra of samples with none or one interferent. Samples of hemoglobin (hemo) = 1190 mg/dL, bilirubin (bili) = 39.65 mg/dL, triglycerides (trig) = 984 mg/dL, and no interference are labeled as 700, 070, 007, and 000, respectively (Table 1).

Hemolysis, icterus, and lipemia are indicated by different features in the absorption spectra that may interfere with one another [1]. Hemolysis is caused by the lysis of blood cells before the cell/supernatant separation to obtain plasma or serum, and hemolysis is quantified by the concentration of hemoglobin, which absorbs at 340 mm to 440 nm and 540 nm to 580 nm (Figure 1A). Icterus is the interference caused by bilirubin, which absorbs light with a broad peak around 460 nm that strongly interferes with the major hemoglobin peak at 415 nm (Figure 1B). Lipemia is the interference caused by lipid particles, which scatter light and lead to an apparent absorption across a wide range of the ultraviolet/visible spectrum (400 nm to 800+ nm). Due to their proximity, the hemoglobin and bilirubin peaks partially overlap with each other, and the apparent absorption by triglycerides affects the whole spectrum (Figures 1-2). Many commercial clinical analyzers utilize absorbance values at wavelengths from 340 nm to 800 nm and complicated calibration procedures to account for this issue [1, 3].

**Figure 3:**
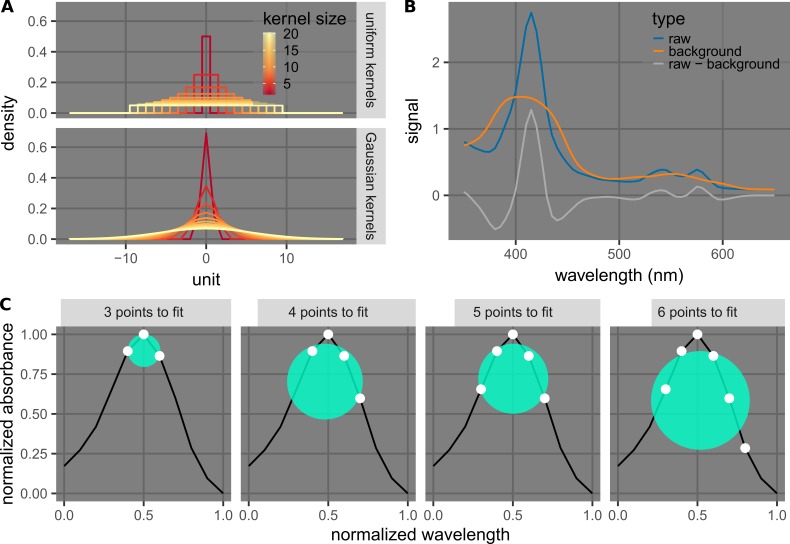
Explanation of the methods of background subtraction (A, B) and curvature calculation (C). A) Plots of possible kernels used for the convolution to calculate the background spectra, which are probability density functions of uniform distributions and Gaussian distributions. The kernel size of a certain distribution function is defined as its standard deviation multiplied by $\sqrt{12}$, which is the range of the distribution if it is uniform. An optimization step may be used to choose an appropriate method specified by the kernel and the wavelength used to obtain the final values. B) Plots showing example “raw,” “background,” and “raw-background” spectra of a sample. The background spectrum was calculated with a uniform kernel of size 14 (14 points * 5 nm/points = 70 nm). C) Plots illustrating curvature calculation. At a certain wavelength (at or near a peak), a certain number of points are used for fitting a circle going through them by minimizing a cost function (Eq. 1). The resulting curvature is the inverse of the radius of such circle. To display the circles with clarity, the wavelength and the absorbance were normalized in these plots, while the actual calculations were done with the original values. An optimization step may be done to choose a curvature calculation method specified by a certain wavelength and number of points used for fitting.

The two new methods described herein (Figure 3A) are intended to eliminate interfering signals and obtain clean spectral signals that enable the quantification of one interferent (hemolysis or icterus) in the presence of various amounts of others. While they are both based on the geometry of the spectra, the details are different.

The first method takes inspiration from background subtraction techniques used in image processing [4] and is more general than those previously employed [5-13]. The processed signal is called the background-subtracted signal. It is obtained by convoluting the raw signal with a blurring kernel to calculate the background (Figure 3A), and subsequently, subtracting the background from the raw signal (Figure 3B).

The second method is derived from the empirical observation that the shapes of the peaks generally do not depend on the background. In this case, we took the processed signal as the spectral curvature near or at the peak of interest, which is the inverse of the radius of the circle fitted through spectral points (Figure 3C). Derivatives, which also provide shape information, have previously been employed to detect and measure hemolysis and icterus [5, 14-19]. Herein, the curvature, which can be calculated using the first and second derivatives, provides a direct description of the shape of the curve near or at a particular peak.

The methods were evaluated with 510 samples containing permutations of levels of hemolysis, icterus, and lipemia, as specified by the concentrations of hemoglobin, bilirubin, and triglycerides, respectively (Table 1). We used this sample set to explain the two new methods described herein and demonstrate their performance in comparison to traditional methods.

**Table 1:** Levels of hemolysis (H), icterus (I), and lipemia (L) in samples used for the demonstration of the new methods described herein. The digits of each sample label indicate the H, I, and L levels (e.g., sample 517 has H, I, and L levels of 5, 1, and 7, respectively). There were a total of 510 samples (8^3^ = 512 permutations, minus samples 577 and 673 lost due to processing errors).

Level	Hemolysis: hemoglobin (mg/dL)	Icterus: bilirubin (mg/dL)	Lipemia: triglycerides (mg/dL)
0	0	0.18	76
1	30	2.76	127
2	50	4.77	175
3	70	9.62	215
4	180	14.63	275
5	370	19.20	462
6	760	29.67	740
7	1190	39.65	984

## Materials and methods

Analytical instruments

The total bilirubin and triglyceride concentrations were obtained on a Siemens ADVIA® 1800 analyzer (Siemens, Munich, Germany). The abbreviations of the total bilirubin and triglycerides assays on the instrument were TBIL 2 and TRIG 2, respectively.

Hemoglobin concentrations (0 to 3,000 mg/dL) were obtained on a HemoCue® Plasma/Low Hb instrument (HemoCue America, Brea, CA, USA) after filtration through 20-µm membranes (Cole-Parmer, Vernon Hills, IL, USA; catalog # EW-32815-00).  Hemoglobin concentrations higher than 3,000 mg/dL were measured on a HemoCue® Hb 201+ instrument (HemoCue America, Brea, CA, USA) without filtration.

Absorption spectra (250 nm to 800 nm, steps of 5 nm) of the samples were acquired on a SpectraMax® M5 spectrophotometer (Molecular Devices, Sunnyvale, CA, USA) at 37°C. All samples were diluted 10 times in saline (0.90% weight/volume) (Thermo Fisher Scientific, Waltham, MA, USA; catalog # 062-125), and dispensed into 384-well plates (Corning, Corning, NY, USA; catalog # 3655) at 30 µL per well.

All measurements were performed in duplicate unless it is otherwise specified.

Sample preparation

Samples were prepared to contain varying levels of hemolysis, icterus, and lipemia. Eight levels of each interferent (labeled 0 to 7) were used to make 512 permutations in total. The sample IDs denote the hemolysis (H), icterus (I), and lipemia (L) levels, respectively (Table 1). Samples 577 and 673 were discarded due to processing errors, so 510 samples were included in the analysis.

The samples were made following the steps described below. All intermediate solutions and samples were frozen and stored at -80°C after each step. They were thawed and brought to room temperature immediately before being used in the subsequent step.

Preparation of Clarified Plasma and Super-Stocks

The clarified plasma was prepared from pooled lithium heparin human plasma obtained from normal healthy individuals (stocked pooled plasma obtained from Access Biologicals, Vista, CA, USA). The plasma was thawed and clarified by centrifugation (7,000 g, 20 minutes, 4°C), 3x paper filtration (grade-691 glass fiber; VWR, Radnor, PA; catalog # 28297-289), and final vacuum filtration with a 0.2-µm membrane (Nalgene, Rochester, NY, USA; catalog # 567-0020). The concentrations of hemoglobin, bilirubin, and triglycerides were 0, 0.19, and 81 mg/dL, respectively.

The hemolysis super-stock was derived from 25 unique human lithium heparin whole blood specimens obtained from normal healthy individuals (specimens obtained from ProMedDx, Norton, MA, USA; IRB approval from New England Independent Review Board (NEIRB)). The specimens were received within three days of collection and processed within one day of receipt. The cells were separated from the plasma by centrifugation at 1,500 g for five minutes, then lysed by freezing at -80°C. This hemolysate was thawed and then combined with clarified plasma at a 2:1 ratio, giving a solution with a hemoglobin concentration of 14,450 mg/dL.

The icterus super-stock was made by dissolving 200 mg of conjugated bilirubin (ditaurate, disodium salt) (CalBioChem, San Diego, CA, USA; catalog # 201102) into 20 mL of the clarified plasma. The concentration of bilirubin in this solution was estimated to be 589 mg/dL.

The lipemia super-stock was an Intralipid® emulsion (20% emulsion) (Sigma, St. Louis, MO, USA; catalog # I141-100ML) used without modification. The total concentration of triglycerides was estimated to be 43,900 mg/dL.

Preparation of Hemolysis, Icterus, and Lipemia Stock Solutions (Eight Levels/Type)

Per each interferent (hemolysis, icterus, or lipemia), eight stock solutions (i.e., “stocks”) were made by combining the corresponding super-stock with the clarified plasma at different ratios. The ratios were determined so that the concentrations in the stocks were targeted to be approximately three times the final concentrations (Table 1).

Preparation of Final Samples with Permutations of Hemolysis, Icterus, and Lipemia Levels

All possible permutations of hemolysis, icterus, and lipemia levels (eight levels/interferent) were prepared using a Hamilton MicroLab STAR liquid handler (Hamilton, Reno, NV, USA). Each sample was prepared by mixing equal volumes of three types of stock solutions described above. Samples 577 and 673 were lost during this process when the pipetting got disrupted unexpectedly. In the end, there were 8^3^ - 2 = 510 samples in total.

**Figure 4:**
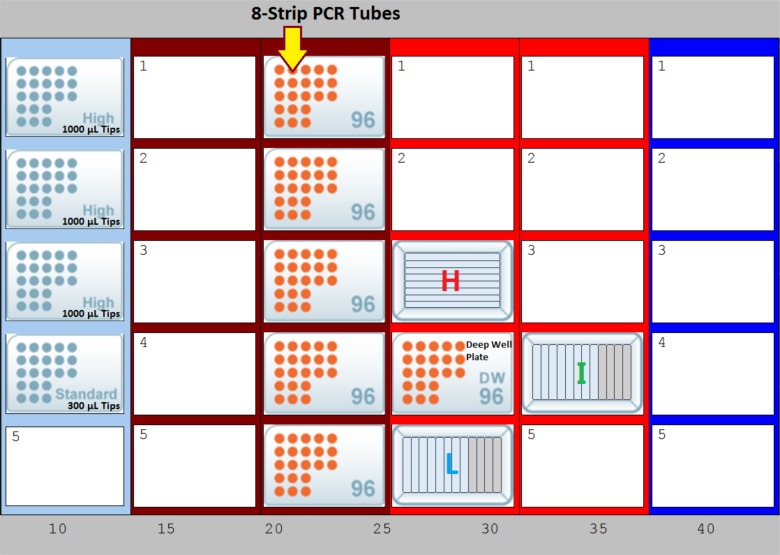
Overview of the setup on the instrument deck of the Hamilton MicroLab STAR liquid-handling platform. Hemolysis (H), icterus (I), and lipemia (L) labels were used to identify the locations of the stocks. Equal volumes from each reservoir (H, I, and L) were combined in the 96-deep well plate.

Hemolysis stocks were dispensed into an eight-row reagent reservoir (deep well, divided, V-bottom) (E&K Scientific, Santa Clara, CA, USA; catalog # EK-2032-S) in increasing concentrations of hemolysis in each row. The eight icterus stocks were dispensed into the first eight columns of a 12-column reagent reservoir (deep well, divided, V-bottom) (E&K Scientific, Santa Clara, CA, USA; catalog # EK-2034-S), in an increasing concentration of bilirubin. Each of the eight lipemia stocks was dispensed into its separate 12-column reagent reservoirs. Equal volumes (600 µL) from each reservoir (H, I, and L) were combined in a 96-deep well plate (Axygen, Corning, NY, USA; catalog # P-DW-20-C-S). After mixing the samples, the automated liquid handler transferred the solutions into eight-strip polymerase chain reaction tubes (E&K Scientific, Santa Clara, CA, USA; catalog # 490048) in 200-µL aliquots. The layout of items on the liquid handler is provided in Figure 4.

While concentrations of interferents of all samples were measured, the true values are based on those of samples with one interferent only. For example, using the naming convention stated above (Table 1), the bilirubin concentration of sample 251 is considered to be the same as that of sample 050.

Data processing and analysis

Signal Calculation

As part of the calculation of background-subtracted signals, the convolution of the original signals with blurring kernels was performed to obtain the background signals. A specific kernel is a probability density function (PDF) (mean, µ = 0; standard deviation, σ). The size of the kernel is defined as σ√12 (e.g., the range, in the case of a uniform distribution). Because the spectra were sampled at discrete wavelengths, the kernels were represented as discrete points along the continuous curves (the PDFs) in the calculation. In particular, for each Gaussian kernel, only points in the [-3σ, 3σ] (rounded) range were used.

The curvature at a specific region of a spectrum is defined as $1/R$, where $R$ is the radius of the circle fitted through the data points in that region. The fitting was done by minimizing the cost function (see Eq. 1 below), where $n$ is the number of points used for fitting, $\vec{X_i}$ is the coordinate (wavelength, absorbance) of point *i*, and $\vec{M}$ is the coordinate of the center.

$$\mathrm{Cost} = \sum_{i=1}^{i=n} \left(\left\| \vec{M}-\vec{X_i}\right\| -R \right) ^{2}$$

Linear Regression

We used linear regression models to evaluate different methods of hemolysis and icterus measurements. In such a model, Y, the quantity of interest (e.g., hemoglobin or bilirubin), is a linear combination of signals from the samples (see Eq. 2 below).

$$Y = a_{0} + a_{1} \mathrm{Signal}_{1} + a_{2} \mathrm{Signal}_{2} + ... + a_{n} \mathrm{Signal}_{n}$$

The model can be trained (calibrated) using a set of samples with known true Ys. In such a process, the coefficients (*a_i_*'s) are varied to minimize the sum of the squared differences between the true Ys and the calculated Ys. The optimized coefficients can then be used to calculate the Ys of the test samples. The signals may be absorbance values at specific wavelengths, differences of absorbance values at two specific wavelengths, the background-subtracted signals, or the curvatures. Models with the newly derived signals were compared with models used in commercial analyzers as described in the literature [1, 3, 20].

Software Tools

The data were processed on a laptop computer using R 3.4.0 run on RStudio 1.0.143 (RStudio, Boston, MA, USA) using packages in the default installation. The function “optim” (in the “stats” package) was used to fit circles through points. The function “filter” (in the “stats” package) was used to calculate the background signals. The function “t.test” (in the “stats” package) was used to perform the Welch’s t-tests. Gaussian kernels (mean, µ = 0, standard deviation, σ) were calculated with the function “dnorm” (in the “stats” package). The function “lm” (in the “stats” package) was used to perform linear regression.

## Results

Example results with background subtraction

**Figure 5:**
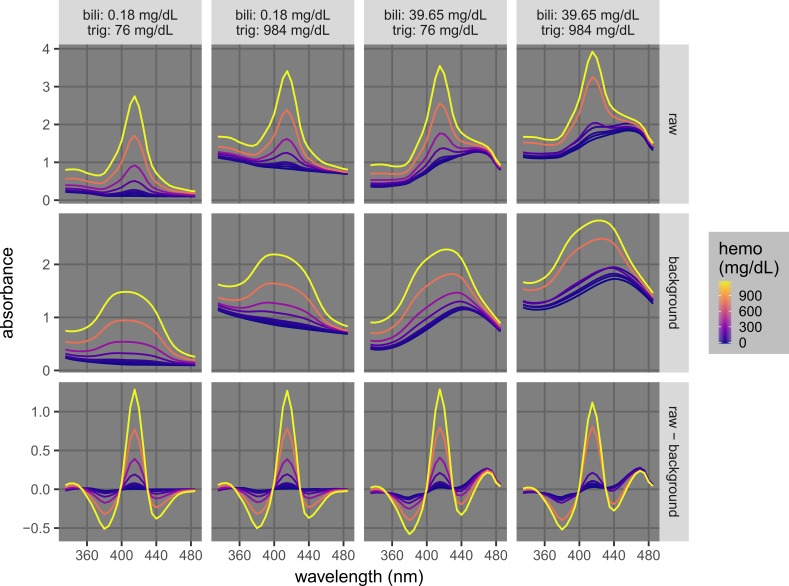
Example background-subtracted spectra used for the quantification of hemolysis. The plots show raw, background, and background-subtracted spectra of samples at different hemolysis levels (hemoglobin [hemo] = 0, 30, 50, 70, 180, 370, 760 and 1190 mg/dL) at permutations of low and high icterus and lipemia (bilirubin [bili] = 0.18 and 39.65 mg/dL; triglycerides [trig] = 76 and 984 mg/dL). The raw spectra were acquired at 5-nm intervals. The background spectra were calculated by convoluting the raw spectra with a uniform kernel of size 14 (or (14-1)*5 = 65 nm; Figure 3A).

We first tested whether background-subtracted spectra can be utilized for the quantification of hemolysis. The accuracy of this approach was demonstrated by the analysis of samples at various levels of hemolysis (hemoglobin = 0, 30, 50, 70, 180, 370, 760, and 1,190 mg/dL) while in the presence of low and high levels of icterus (bilirubin = 0.18 and 39.65 mg/dL) and lipemia (triglycerides = 76 and 984 mg/dL) (Figure 5). Without the background subtraction step, the raw absorption spectra contained unwanted signals from icterus and lipemia, and the peaks at 415 nm were elevated. Background-subtracted spectra with different levels of icterus and lipemia were similar in the region of interest. For example, with the uniform kernel of size 14 (1 unit = 5 nm), the background-subtracted absorbance values at 420 nm at different hemolysis levels were similar in all interference conditions tested and were not dependent on the levels of icterus and/or lipemia.

**Figure 6:**
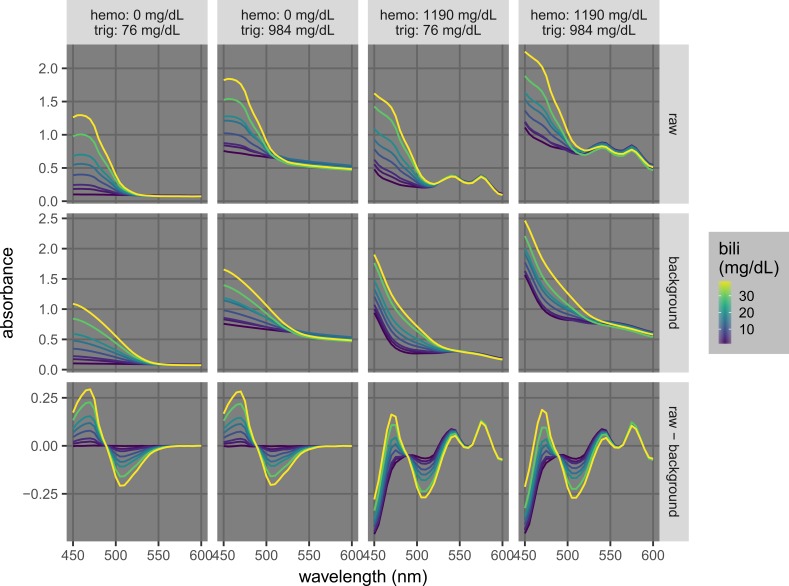
Example background-subtracted spectra used for the quantification of icterus. The plots show raw, background, and background-subtracted (raw-minus-background) spectra of samples at different icterus levels (bilirubin [bili] = 0.18, 2.76, 4.77, 9.62, 14.63, 19.20, 29.67, and 39.65 mg/dL) at permutations of low and high hemolysis and lipemia (hemoglobin [hemo] = 0 and 1,190 mg/dL; triglycerides [trig] = 76 and 984 mg/dL). The raw spectra were acquired at 5-nm intervals. The background spectra were calculated by convoluting the raw spectra with a uniform kernel of size 17 (or (17-1)*5 = 80 nm; Figure 3A).

Similarly, background-subtracted spectra can be utilized for the quantification of icterus (Figure 6). The bilirubin concentrations were 0.18, 2.76, 4.77, 9.62, 14.63, 19.20, 29.67, and 39.65 mg/dL; the hemoglobin concentrations were 0 and 1,190 mg/dL; and the triglyceride concentrations were 76 and 984 mg/dL. While the raw spectra were significantly affected by hemolysis or lipemia, the background-subtracted spectra were similar in all interference permutations tested. In particular, with the uniform kernel of size 17 (1 unit = 5 nm), the background-subtracted absorbance values at 525 nm were not affected by the levels of hemolysis and/or lipemia.

Example results with curvature calculation

**Figure 7:**
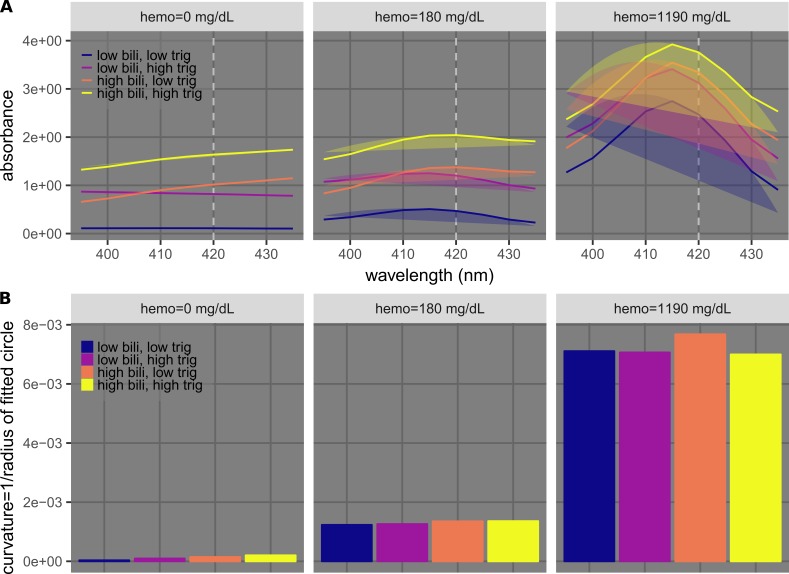
Example results of using curvature calculation to quantify hemolysis. A) Plots showing spectra of samples with three different hemo concentrations (0, 180, 1,190 mg/dL) in separate sub-panels. Each sub-panel shows spectra of samples at low/high permutations of icterus (bilirubin [bili] = 0.18 and 39.65 mg/dL) and lipemia (triglycerides [trig] = 76 and 984 mg/dL). The dashed lines indicate 420 nm. The shaded circular sectors indicate fitted results using four data points around 420 nm (415, 420, 425, and 430 nm). B) Bar charts showing curvatures calculated from results shown in A.

We observed that the shape of a specific absorption peak (e.g., the 415-nm hemoglobin peak) did not change if there was interference by a nearby peak (e.g., the 460-nm bilirubin peak) or by an increase in absorption across a wide range of wavelengths (e.g., in the case of lipemia). This was demonstrated with samples having different hemolysis levels (hemoglobin = 0, 180, or 1,190 mg/dL) and different permutations of low/high levels of icterus (bilirubin = 0.18 and 39.65 mg/dL) and lipemia (triglycerides = 76 and 984 mg/dL) (Figure 7). At each hemolysis level, samples with different icterus and lipemia levels were found to have markedly different absorbance values (Figure 7A). Circles were fitted to points near the peaks (at 415, 420, 425, and 430 nm) using least-squares regression with the cost function defined in Eq. 1 (Figure 7A). The resulting curvatures (the inverses of the radii), which were used as metrics to quantitatively describe the shapes of the peaks, were similar for each group of samples at each hemolysis level, regardless of icterus and lipemia levels (Figure 7B). This result demonstrates the possibility of using the curvatures to quantify and detect hemolysis.

**Figure 8:**
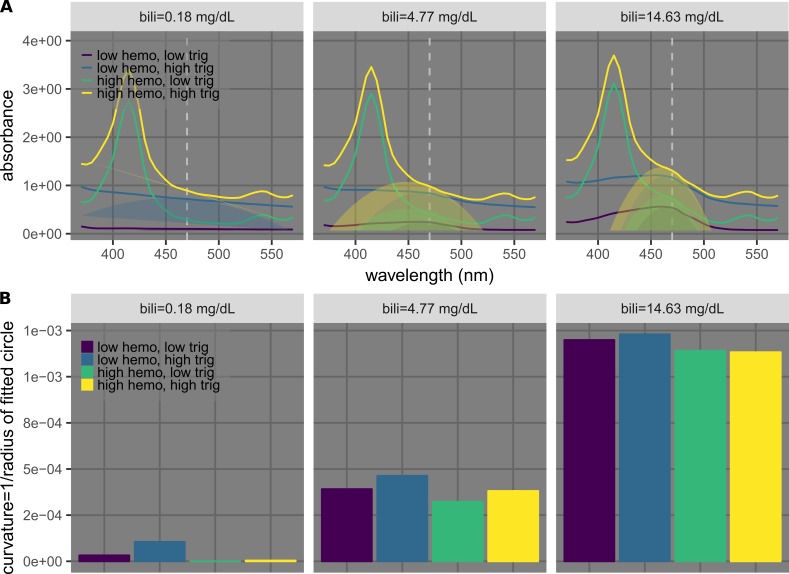
Example results of using curvature calculation to quantify icterus. A) Plots showing spectra of samples with three different bilirubin (bili) concentrations (0.18, 4.77, 14.63 mg/dL) in separate sub-panels. Each sub-panel shows spectra of samples at low/high permutations of hemolysis (hemoglobin [hemo] = 0 and 1190 mg/dL) and lipemia (triglycerides [trig] = 76 and 984 mg/dL). The dashed lines indicate 470 nm. The part-circles indicate fitted results using four data points around 470 nm (465, 470, 475, and 480 nm). B) Bar charts showing curvatures calculated from results shown in A.

This method was also applied to icterus detection and quantification. Similar to the case of hemolysis (Figure 7), samples at three different levels of icterus (bilirubin = 0.18, 4.77, and 14.63 mg/dL) and different low/high permutations of hemolysis (hemoglobin = 0 and 1,190 mg/dL) and lipemia (triglycerides = 76 and 984 mg/dL) were used to demonstrate feasibility (Figure 8). At each icterus level, samples with different hemolysis and lipemia levels were found to have markedly different absorbance values (Figure 8A). Circles were fitted to data points near the peaks (at 465, 470, 475, and 480 nm) using least-squares regression with the cost function defined in Eq. 1 (Figure 8A). The resulting curvatures (the inverses of the radii), which we used as metrics to quantitatively describe the shapes of the peaks, turned out to be similar for each group of samples at each hemolysis level, regardless of icterus and lipemia levels (Figure 8B).

Optimization of parameters

There are multiple options to consider when applying the new methods described herein. The background calculation step can be done with different kernels of different types and sizes and requires specification of the wavelength of interest to obtain the processed signal. The curvature calculation can be done with different choices for the center wavelength and a different number of points around each chosen center wavelength. In addition, there are multiple metrics to evaluate different implementations, and we considered two metrics herein. The first metric is the R^2^ obtained from fitting the reference concentrations with the calculated signals. The second is the p-value of the Welch’s t-test performed on two groups of samples of the lowest and second lowest levels, which is motivated by the possible need for very sensitive detection in some applications [21]. For practicality, -log_10_(p) values were used instead of p-values. Note that the Welch’s t-test was chosen over the student’s t-test because the variances at different levels are not expected to be the same.

**Figure 9:**
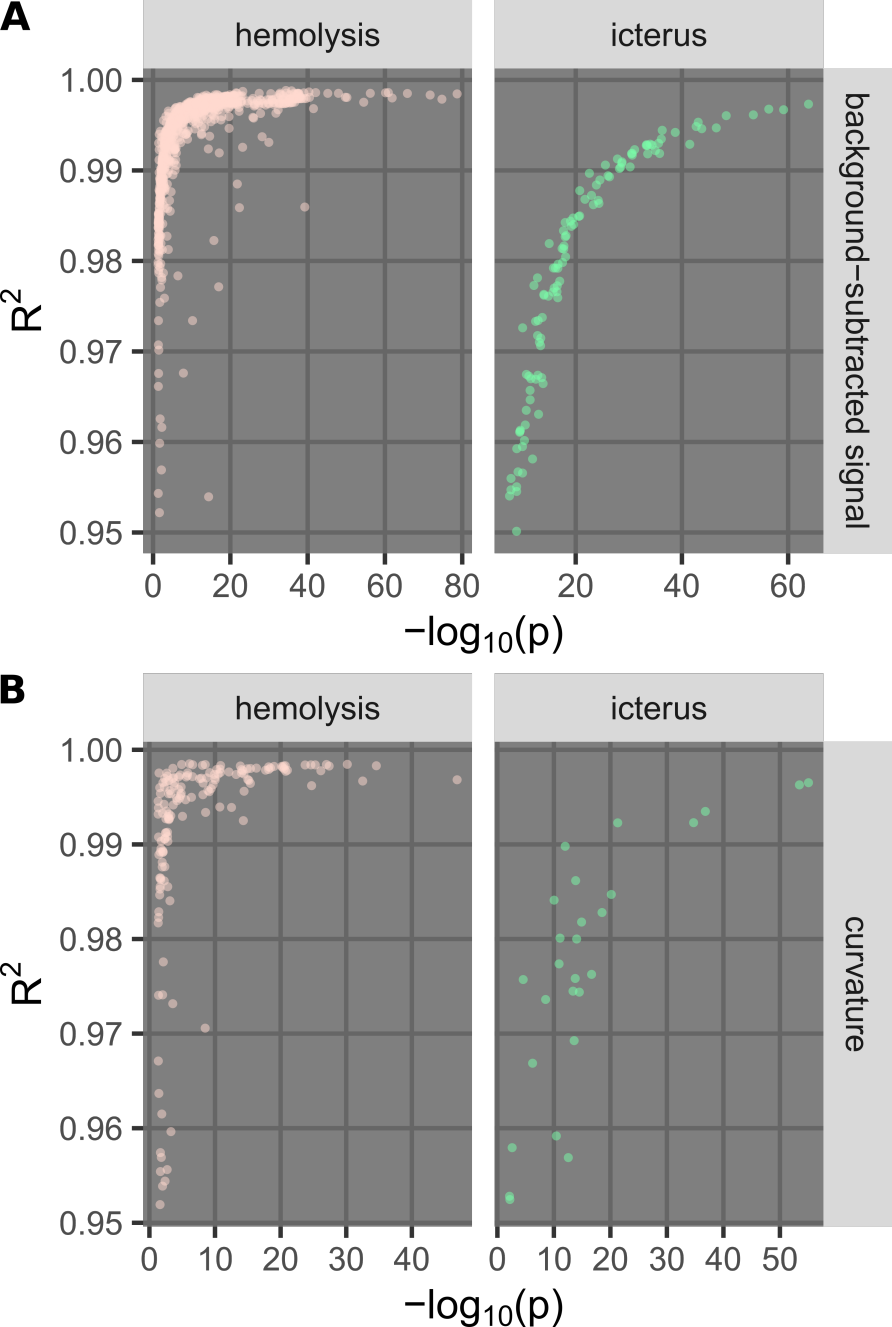
Comparison of metrics used for parameter optimization. The plots show metrics calculated from using the background subtraction (A) and curvature calculation (B) methods to quantify and detect hemolysis and icterus over a large parameter space. For a certain method with a certain parameter set, the R^2^ value was calculated from fitting the calculated signal (background-subtracted signal or curvature) with the concentration (hemoglobin or bilirubin), and the -log_10_(p) value was calculated from the p-value of the Welch’s t-test performed on two groups of samples, one with the lowest level and one with the second highest level of the specific interferent (Table 1). The better the method, the higher the R^2^ value or the higher the -log_10_(p) value. While all points from the parameter search were calculated, for clarity, the plots were zoomed in with R^2^ ≥ 0.95 and -log_10_(p) ≥ 1.3 (i.e., p ≤ 0.05).

We performed an optimization step to determine the optimal parameters for each method (background subtraction or curvature calculation) for each interferent (hemolysis or icterus). In particular, the center wavelength was varied from 350 nm to 650 nm (with steps of 5 nm). For background calculation, the kernel types were 1) Gaussian and 2) uniform, while the size was varied from 2 to 20 units (1 unit = 5 nm). For the curvature calculation, the number of points was varied from three to 16. At a certain center wavelength, if the number of points was even, more points were chosen on the side of larger wavelengths. Metrics of R^2^ and -log_10_(p) for all cases were calculated. Only points with good overall performance (R ≥ 0.95 and p ≤ 0.05 ⇔ -log_10_(p) ≥ 1.3) were plotted (Figure 9). The metric -log_10_(p) was chosen because of its usefulness; if a method has a high -log_10_(p), it also has a high R^2^, while the converse is not true.

**Figure 10:**
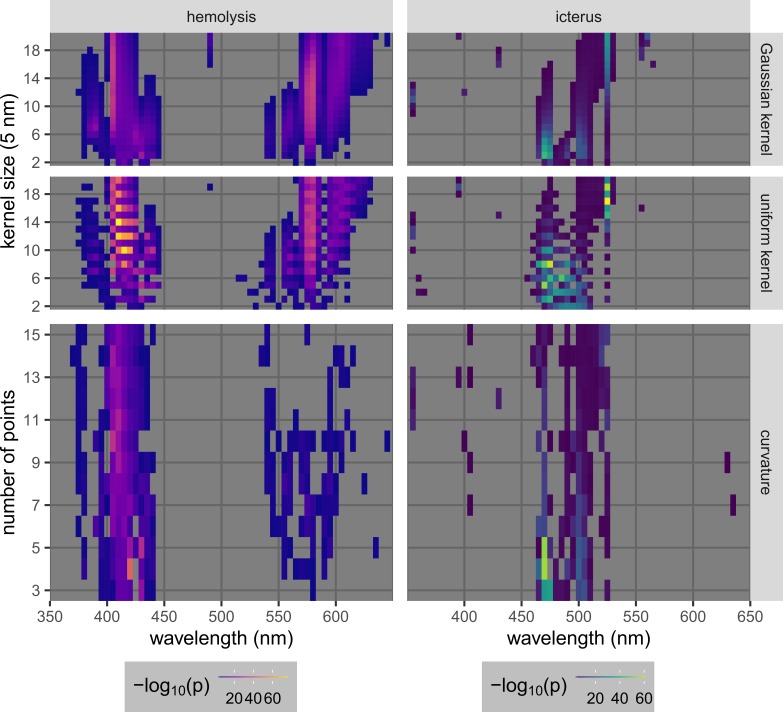
Optimization results for the quantification and detection of hemolysis and icterus, using either background subtraction (with Gaussian kernels or uniform kernels) or curvature calculation. The heat maps show results over wide ranges of parameters, which were the center wavelength selected to generate the processed signal, the kernel size (in the case of background subtraction), and the number of points used for curve fitting (in the case of curvature calculation). The metric used for the optimization was -log_10_(p) calculated from the p-value of the Welch’s t-test performed on two groups of samples, one with the lowest level and one with the second highest level of each interferent (Table 1). Only points with good performance (p ≤ 0.05 ⇔ -log_10_(p) ≥ 1.3) are plotted.

Optimization results, as expressed in heat maps (Figure 10), showed that optimal wavelengths are near the peaks of the spectra of the substances of interest (415, 540, and 575 nm for hemoglobin, and 460 nm for bilirubin) (Figure 1). This was expected since the peaks are normally used as the signatures of the corresponding spectra. The optimal parameter sets indeed included wavelengths near the expected peaks (Table 2). There were also wide ranges of values of parameters (wavelength, kernel type/size, and the number of points for fitting) that gave good results (p-value ≤ 0.05). However, it was apparent that methods involving background subtraction with uniform kernels are generally better than those using Gaussian kernels and those with curvature calculation, for both hemolysis and icterus cases.

**Table 2:** Parameter search (Figure 10) results for hemolysis and icterus detection using background subtraction and curvature calculation, in comparison to the raw signals (raw absorbance values). The selected parameters are those that provided the highest –log_10_(p).

	Hemolysis	Icterus
Raw signal	415 nm (raw absorbance values)	460 nm (raw absorbance values)
Background-subtracted signal	410 nm, size-14 uniform kernel	525 nm, size-17 uniform kernel
Curvature	4 points about 420 nm	4 points about 470 nm

Performance of the new methods

**Figure 11:**
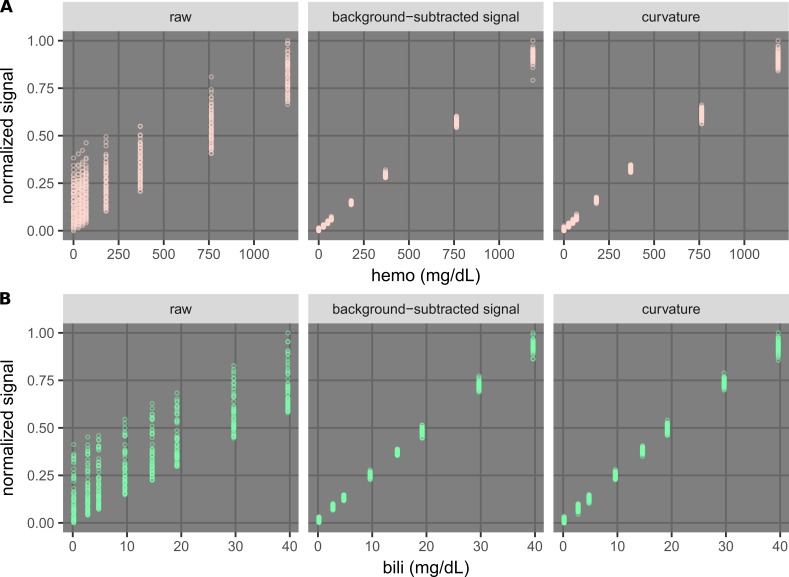
Qualitative comparison of the background-subtracted signals and the curvatures with the raw signals. A) Plots showing hemolysis signals versus the hemoglobin (hemo) concentration. The raw signal is the absorbance at 415 nm. The background-subtracted signals were obtained with parameters of 410 nm and a size-14 uniform kernel, and the curvatures were calculated at 420 nm using four points (Figure 10). B) Plots showing bilirubin (bili) signals versus the bili concentrations. The raw signal is the absorbance at 460 nm. The background-subtracted signals were obtained with parameters of 525 nm and a size-17 uniform kernel, and the curvatures were calculated at 470 nm using four points (Figure 10). In each plot, the signals were normalized for the purpose of visual comparison.

A qualitative comparison of the different types of signals (Table 2) was done using plots of normalized signals versus concentrations for all samples (Figure 11). For the purpose of comparison, the signals of each plot were linearly normalized so that the lowest signal was 0 and the highest was 1. In both hemolysis and icterus cases, the raw signals at each concentration were spread out over a large range (0.3 to 0.4 in the normalized scale), indicating that quantification and detection using the raw signal would be almost impossible. On the other hand, points in the plots of the background-subtracted signals and the curvatures collapsed into much smaller ranges (resembling lines), improving the correlations.

**Figure 12:**
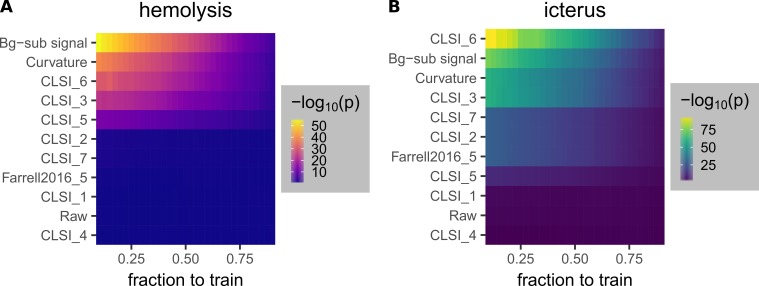
Comparison of the performance of models used to quantify and detect hemolysis (A) and icterus (B). The models are linear regression models described above (Eq. 2, Table 3). The fraction to train indicates the ratio of the number of samples randomly chosen to train versus the total number of samples (Table 1). At each point on the heat maps, the color corresponds to the -log_10_(p) value obtained from the Welch’s t-test performed on the Ys (Eq. 2) of two groups of samples of the lowest and second lowest interference levels, averaged over 10 different iterations.

We compared the performance of each of the optimized methods (Table 2) to those of traditional methods currently used in conventional chemical analyzers [1, 3, 20] using linear regression models. Each model (Table 3, Eq. 2) was evaluated by 10 iterations at each training fraction. In each iteration, the sample set (Table 1) was randomly split into training and testing sets, with the training fraction specifying the ratio of the number of samples in the training set versus the total number of samples. The metric calculated from each iteration was the same as the metric used to optimize the new methods (Figure 9), which is the -log_10_(p) value obtained from the Welch’s t-test performed on two groups of samples of the lowest and second lowest interferent levels. The -log_10_(p) values were averaged over the iterations. The results for both hemolysis (Figure 12A) and icterus (Figure 12B) showed that the background-subtracted signals and the curvatures performed better than most other models that involve multiple absorbance values (Table 3). In particular, models that use either background-subtracted signals or curvatures were in the top three for both hemolysis and icterus detection.

**Table 3:** Descriptions of linear regression models used for hemolysis and icterus quantification. The wavelengths were rounded to multiples of 5 nm in the implementation. Each ’/’ indicates the signal is the difference between absorbance values at the two specified wavelengths. Each ’;’ character is used to separate multiple signals used in the same model.

Model name	Interferent	Signals (absorbance values or other types)
CLSI 1	Hemolysis	405/700
CLSI 1	Icterus	452/700
CLSI 2	Hemolysis	571/596
CLSI 2	Icterus	478/505
CLSI 3	Hemolysis	572/604; 628/660
CLSI 3	Icterus	500/524; 572/604; 628/660
CLSI 4	Hemolysis	522/750
CLSI 4	Icterus	507/776
CLSI 5	Hemolysis	410/480; 600/800
CLSI 5	Icterus	480/570; 600/800
CLSI 6	Hemolysis	340; 410; 470; 600; 670
CLSI 6	Icterus	340; 410; 470; 600; 670
CLSI 7	Hemolysis	570/600
CLSI 7	Icterus	480/505
Farrell2016_5	Hemolysis	583/629
Farrell2016_5	Icterus	480/512
Bg-sub signal (Table 2)	Hemolysis	410 nm, size-14 uniform kernel
Bg-sub signal (Table 2)	Icterus	525 nm, size-17 uniform kernel
Curvature (Table 2)	Hemolysis	4 points about 420 nm
Curvature (Table 2)	Icterus	4 points about 470 nm
Raw	Hemolysis	415
Raw	Icterus	460

Advantages of the new methods

The two methods described herein have three major practical advantages versus interference correction methods that are based on absorbance values at multiple wavelengths across the ultraviolet/visible range, such as those used for hemolysis and icterus detection on many commercial analyzers. First, the methods described herein are less susceptible to the presence of unknown interferents. The choice of wavelengths in traditional methods depends on absorption wavelengths of known interferents [1], while both curvature calculation and background subtraction are mostly agnostic of the interferents and only depend on the absorption of the substance of interest. Even though the optimized hemolysis and icterus signals slightly deviated from the peaks (415 nm for hemolysis and 460 nm for icterus) (Table 2), the signals at the peaks would still provide good performance, with p-values distinguishing the two lowest interference levels (of hemolysis or icterus) much lower than 0.05 (i.e., -log_10_(p) values much larger than 1.3) (Figure 10).

**Figure 13:**
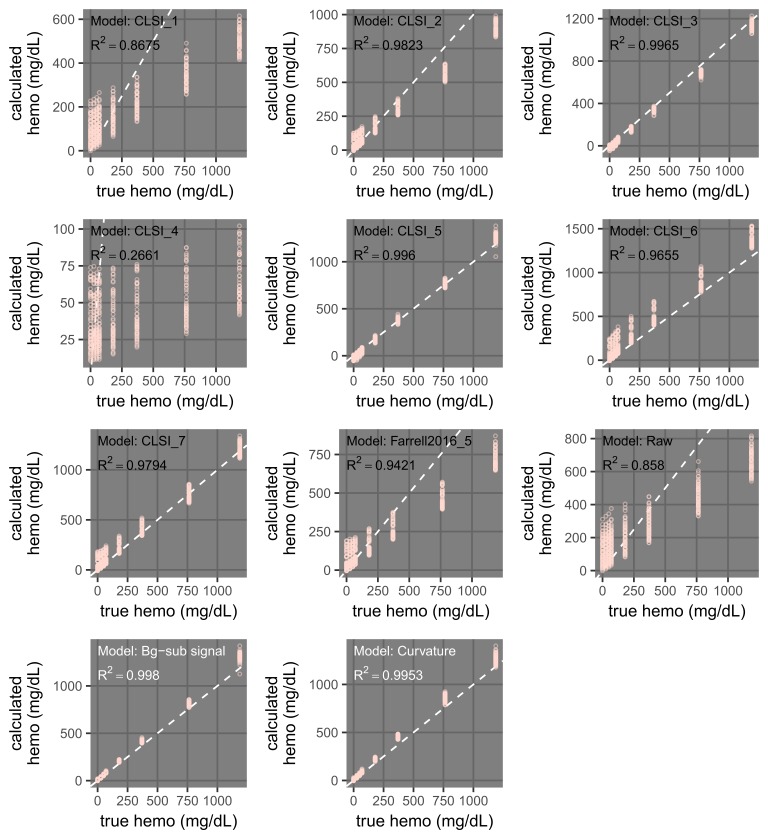
Dependence of the performance of hemolysis regression models on the interference levels used in calibration (training). Plots comparing hemoglobin (hemo) from regression models versus true samples are shown. For calibration, the models used only samples with interference levels of 0 or 1 (total of eight samples). The models are described in Table 3. A subset of these plots are shown in Figure 15A. The dashed white lines indicate where calculated values are equal to true values.

**Figure 14:**
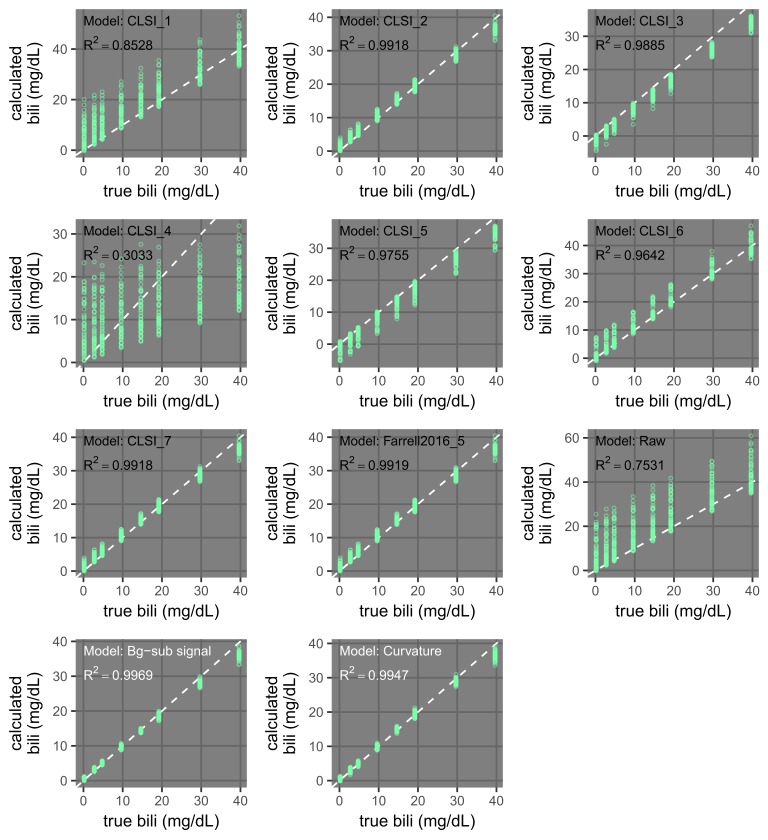
Dependence of the performance of icterus regression models on the interference levels used in calibration (training). Plots comparing bilirubin (bili) calculated from regression models versus true samples are shown. For calibration, the models used only samples with interference levels of 0 or 1 (total of eight samples). The models are described in Table 3. A subset of these plots are shown in Figure 15B. The dashed white lines indicate where calculated values are equal to true values.

**Figure 15:**
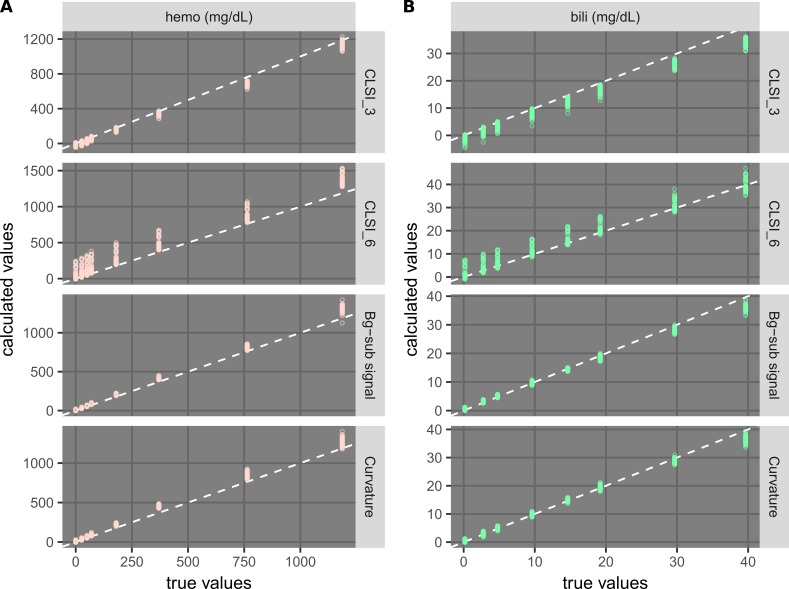
Performance dependence of example regression models on the interference levels used in calibration. Plots comparing concentrations calculated from regression models with true values for hemolysis (A) and icterus (B) are shown. The concentrations were calculated for all 510 samples (Table 1) using calibration information from only eight level-0 and level-1 samples. Results of some example models are shown, while those of all models (Table 3) are provided in Figures 13-14. The dashed white lines indicate where calculated values are equal to true values.

**Figure 16:**
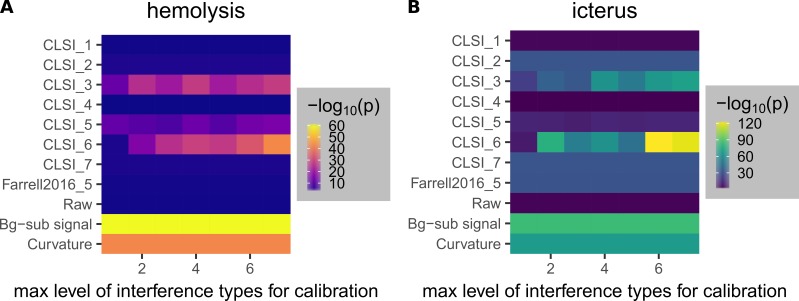
Dependence of regression models on the maximum levels of interference in the samples used for calibration (training). Heat maps of the performance of models to detect hemolysis (A) and icterus (B) at special maximum interference levels in samples used for calibration are shown. The levels of interferents are described in Table 1; the models are described in Table 3. The colors in the heat maps correspond to -log_10_(p) values calculated using the Welch’s t-test performed on two groups of samples of the lowest and second lowest levels (Figure 9).

Second, traditional methods require calibration using samples with wide ranges of interference levels [1], while the methods using background-subtracted signals or curvatures do not. We performed an example analysis to demonstrate this notion. Using only samples with a maximum interference level of 1 (Table 1) to calibrate regression models (Table 3), we calculated the corresponding hemoglobin and bilirubin values for all 510 samples. In the eight samples used for these calibration steps, the highest hemoglobin (30 mg/dL), bilirubin (2.76 mg/dL), and triglyceride (127 mg/dL) levels were practically low. Traditional methods gave large biases and poor correlations, while those using background-subtracted signals and curvatures gave good agreement (Figures 13-15). As expected, with -log_10_(p) as the metric, the maximum level of interference used for calibration had to be increased for the performance of traditional methods to improve. In contrast, the methods using background-subtracted signals or curvatures performed very well, even when a maximum level of 1 is used for calibration (Figure 16). The independence of the methods involving background-subtracted signals and curvatures allows the calibration to be done even with samples of limited interference levels (e.g., those naturally collected instead of those made via a comprehensive procedure like the samples used for this work).

**Figure 17:**
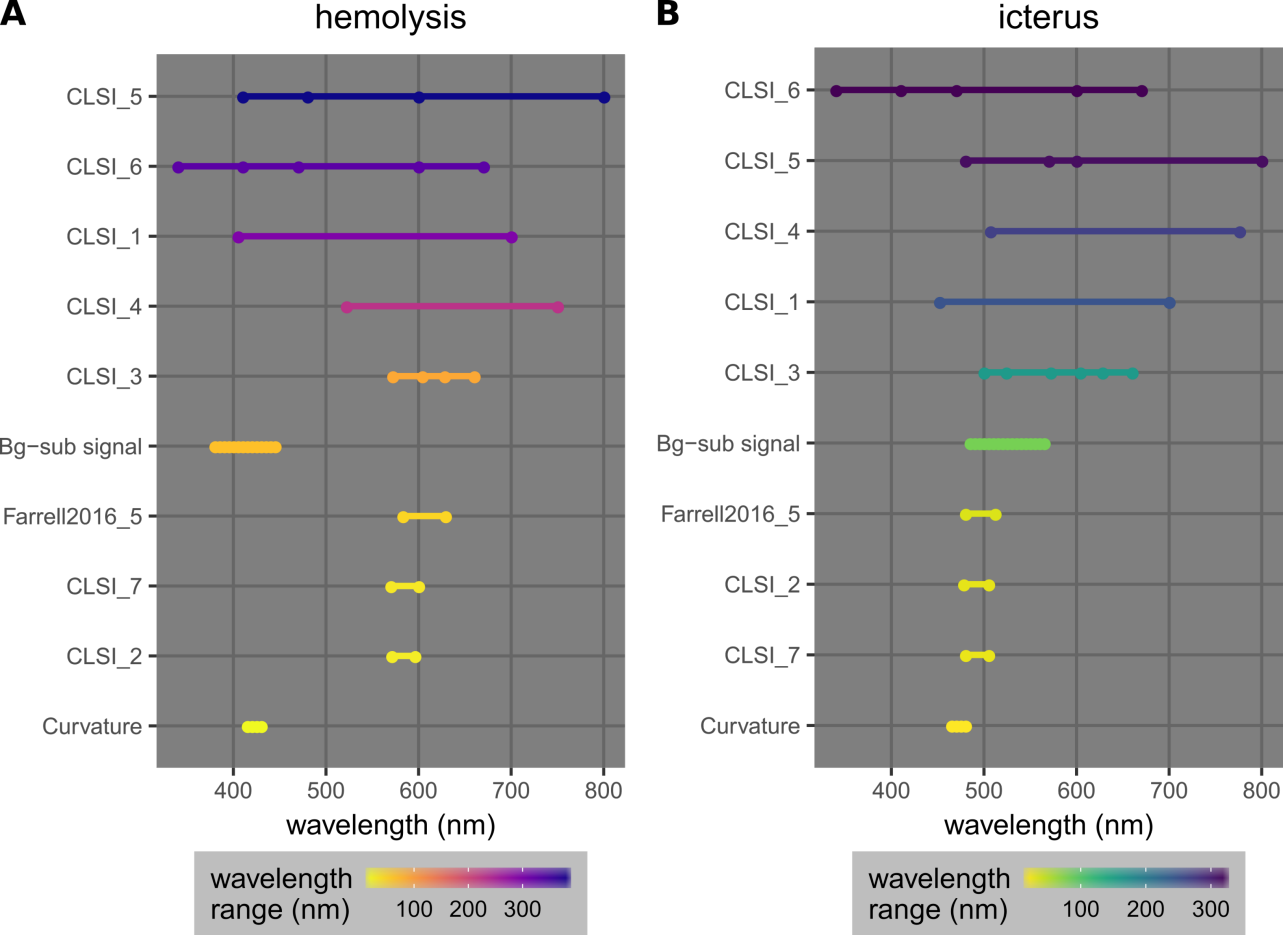
Wavelength ranges required by different linear regression models used for hemolysis (A) or icterus (B) quantification (Table 2). The models are sorted by the sizes of the wavelength ranges.

Third, only a small range of wavelengths around the major wavelength of interest is required for both new methods (Figure 17). In particular, optimized curvatures require only a 15-nm wavelength range, the shortest for both hemolysis and icterus cases.

Background-subtracted signals require ranges spanning 65 nm for hemolysis and 80 nm for icterus, which are ranked in the middle in both cases. This advantage enables possible simplifications and improvements of the detection instrument, such as a smaller wavelength range of the light source, a smaller required width of the array detector (in a prism/grating-based spectrophotometer), and a higher wavelength resolution (due to a smaller wavelength range for the same detector size). While typical commercial analyzers have full-scale spectrophotometers, point-of-care devices may benefit from this advantage.

## Discussion

The key approach of the two new methods described herein (those involving background-subtracted signals and curvatures) is to obtain clean spectral signals even with interference. Therefore, their application can be extended to other spectral measurements. For example, many clinical assays with optical readouts based on absorption spectra employ multiple-wavelength readings to subtract out interfering signals and require the knowledge of possible interferents [13]. The two new methods described can be readily applied to those assays, with the practical advantages described above.

It is worth noting that the background-subtracted signals may be negative at certain points in the parameter space (as specified by the wavelength, kernel type, and kernel size) (Figures 5-6). A negative signal may occur at a wavelength next to a peak or at a peak near another one that is much higher. At first glance, it may not seem intuitive to use such negative signals, but they do contain information about the peaks of interest. Indeed, the optimal background-subtracted signal for icterus measurements (Table 3) was negative (Figure 6) but still performed well (Figures 11B, 12B, 15B).

**Figure 18:**
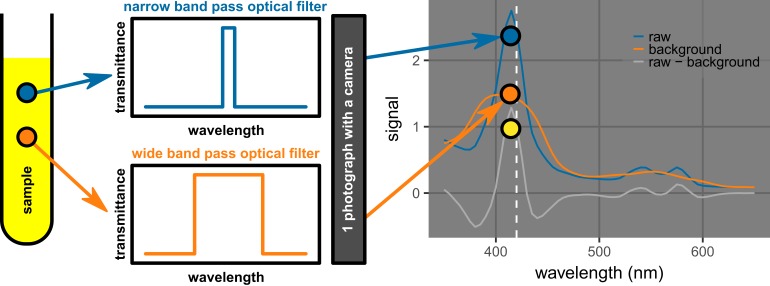
Proposed method of using photography to acquire background-subtracted signals with a narrow band pass optical filter and a wide band optical filter. If the two filters can be placed in the regions of interest concurrently, only one exposure is needed to obtain the major signal and the background signal. The background-subtracted signal can then be readily calculated. The bands are centered near or at the absorption peak of interest.

In some settings, such as when hemolysis detection is required at the collection site, the use of an inexpensive and simple device to collect spectral data is desirable. The background subtraction method described herein can potentially enable such data acquisition to be done with a simple camera. The novelty is that the background subtraction method only requires two optical filters, one with a narrow band to obtain the major signal, and the other one with a wider band to obtain the background signal (Figure 18). If the two optical filters are placed in the region of interest side-by-side in the same field of view, only one image is needed to obtain the background-subtracted signal for each sample. Such a design would be compatible with resource-limited settings. For example, to detect hemolysis at the collection site without a spectrophotometer, one could photograph the plasma fractions of centrifuged collection devices using a simple point-and-shoot camera (or a cell phone camera) with the light path that includes a hybrid filter, which is composed of a narrow-band pass region and a wide-band pass region (e.g., 5-nm and 65-nm wide filters for hemolysis measurements). There have been efforts to develop technologies to detect hemolysis at the collection site such as one involving the use of color intensities of images taken with a camera [22]. The method proposed herein (Figure 18) would require a much smaller and simpler sample set for calibration and provide performance similar to that of a spectrophotometer, thus avoiding the need to deal with the complex, non-trivial conversion of the absorption/scattering spectra to recorded colors.

## Conclusions

The new methods described in this paper, which are based on background-subtraction and curvature calculation, provide the ability to quantify and detect hemolysis and icterus with several practical advantages: 1) better robustness in terms of eliminating signals from unwanted substances, some of which may not be known beforehand, 2) smaller sets of samples used for calibration with few levels of interference, and 3) simpler instruments (spectrophotometers with smaller detectors/short wavelength ranges or cameras equipped with pairs of filters). These new methods do not have advantages over traditional methods with respect to the number of discrete wavelengths required. A camera-based implementation would require further hardware engineering, and the implementation of these new methods, in general, may involve other methods of performing background-subtraction (e.g., those with other blurring methods) or curvature calculation (e.g., those using methods other than circle fitting). Such implementation could benefit cases of sample collection in resource-limited settings. For example, in remote sites where samples are collected and sent to centralized laboratories, the ability to detect interference at the point of collection would allow for immediate re-drawing. Furthermore, if the hardware is adapted to work with small-volume samples (e.g., those collected by fingersticks), it would be possible to integrate the methods described herein with point-of-care diagnostic instruments and contribute to the effort of bringing diagnostics to developing countries or other under-served settings. Overall, these new data analysis methods can enable new practical possibilities in the development of interference screening methods.
